# Examining the Impact of Key Factors on COVID-19 Vaccination Coverage in India: A PLS-SEM Approach

**DOI:** 10.3390/vaccines11040868

**Published:** 2023-04-19

**Authors:** Veena Dhawan, Mahesh Kumar Aggarwal, Pritu Dhalaria, Preeti Kharb, Deepika Sharma, Kamala Kannan Dinesh, Sanjay Dhir, Sushil, Gunjan Taneja, Raj Shankar Ghosh

**Affiliations:** 1 Ministry of Health & Family Welfare, Government of India, New Delhi 110011, India; 2 Immunization Technical Support Unit, Ministry of Health & Family Welfare, Government of India, New Delhi 110011, India; 3 Department of Management Studies, Indian Institute of Technology Delhi, New Delhi 110016, India; 4 Bill & Melinda Gates Foundation, New Delhi 110067, India; 5 Public Health Consultant, New Delhi 110016, India

**Keywords:** COVID-19, government stewardship, partial least square, community participation, vaccination coverage, India

## Abstract

During the coronavirus disease 2019 (COVID-19) pandemic, numerous factors determined the performance of COVID-19 vaccination coverage. The purpose of this study is to examine the influence of factors such as government stewardship, planning and implementation, and community participation on COVID-19 vaccination coverage. This study applied partial least square structured equation modeling (PLS-SEM) by analyzing 187 responses from the stakeholders involved in vaccination programs in four select states of India. This study empirically validates a framework for improving vaccination coverage by confirming the significant impact of planning and implementation on vaccination coverage followed by government stewardship and community participation. Additionally, this study highlights the individual impact of each factor on vaccination coverage. Based on the findings, strategic recommendations were proposed that can be utilized for formulating policy-level actions to facilitate the vaccination program.

## 1. Introduction

Vaccination is a crucial public health intervention to protect the population from life-threatening diseases, including COVID-19 [1,2]. Despite the rate at which safe and effective vaccines were developed, vaccination coverage is still a matter of concern across the world [3]. In a developing country such as India, which amounts to one-seventh of the total global population of around 1.3 billion people, a robust vaccination coverage plan has helped to reduce the surge of disease and get the economy back on track [4]. As COVID-19 is a highly transmissible virus, one of the critical strategies to combat its ill effects is to develop population immunity that can be achieved through high vaccination coverage [5,6,7]. Various vaccines have been given Emergency Use Approval (EUA); however, the prime concern was concerning their implementation at the national, regional, and local levels [8]. India’s nationwide COVID-19 vaccination program was launched on 16 January 2021. The Government of India concentrated all its efforts on ensuring logistic and financial resources available for the production, acquisition, and nationwide distribution of COVID-19 vaccines to control the COVID-19 pandemic [9]. India supported the research, development, and manufacturing of COVID-19 vaccines under the “Make-in-India” and “Make-for-World” Strategy, embarked on the use of cutting-edge technologies such as the COVID-19 vaccine intelligence network (CoWIN) for evaluating geographical coverage, tracking adverse events following immunization (AEFI) for vaccines, promoting inclusivity, and for providing a single reference point for citizens to follow their vaccination schedule, and prioritized vaccine administration based on scientific evidence and global best practices.

Several systematic interventions were also carried out in ensuring capacity building for carrying out this nationwide exercise. The existing supply chain for storage and transport of COVID-19 vaccines was leveraged and strengthened and effective monitoring of vaccine distribution and assured availability and efficient utilization of vaccines and syringes was ensured at all times. Additionally, the government ensured timely vaccination coverage in a planned manner, starting with front-line workers and a population >60 years in the first phase followed by the coverage of 45–60 years and >18 years’ age group in the third phase. The significance of the critical factors that lead to high vaccination coverage started unfolding. India’s free and voluntary nationwide COVID-19 vaccination exercise is also being carried out in a citizen-friendly approach through initiatives such as Har Ghar Dastak, Workplace COVID-19 vaccination center (CVC), School-based vaccination, vaccination of persons with no identity documents, Near-to-Home CVC, and Mobile Vaccination Teams. With 71% of CVCs located in rural areas and over 51% of vaccine doses administered to women, India’s National COVID-19 Vaccination Program also ensured geographical and gender equity. India also laid out a well-organized communication strategy of providing correct information and customized guidelines on COVID-19 vaccination. It helped address vaccine hesitancy and promoted vaccine eagerness and COVID-19-appropriate behavior among the masses (https://pib.gov.in/PressReleasePage.aspx?PRID=1842157#:~:text=In%20a%20historic%20achievement%2C%20India’s,2%2C63%2C26%2C111%20sessions, accessed on 15 February 2023). 

Despite the decline in COVID-19 cases across the country, consistent efforts were ongoing to vaccinate all eligible citizens. This is exemplified by the fact that it took almost 9 months to reach the 1000 million mark and another 9 months to reach the 2000 million vaccination mark since the start of the vaccination drive on 16 January 2021, with the highest single-day vaccination record of 25 million doses achieved on 17 September 2021. On 15 July 2022, the Union Government launched a 75-day long ‘COVID-19 Vaccination Amrit Mahotsav’ to provide free precaution doses to all eligible adult populations at Government COVID-19 Vaccination Centers (CVCs).

The Union Government, in close collaboration with State Governments/U.T administration has been working over a period of time to ensure that these efforts collectively culminated in the success of COVID-19 vaccination in India. The extant literature has examined the impact of numerous factors on vaccination coverage, including the significant impact of vaccine acceptance. However, most studies have focused on identifying the factors influencing vaccination coverage within a limited segment and context of the population. Therefore, a research gap has been identified in exploring and examining the factors impacting vaccination coverage in a comprehensive and broader context and population. In this study, the rationale behind this broad coverage is analyzed through the lens of the role of government stewardship (both central and state level), planning, and implementation for achieving coverage and active community participation in the COVID-19 vaccination program [10]. These factors have been critically analyzed for four states (Andhra Pradesh, Himachal Pradesh, Maharashtra, and Orissa) in India where there was significantly high vaccination coverage. The following subsections showcase the theoretical background of these identified factors and the proposed hypotheses for this study.

### 1.1. Government Stewardship

The extant literature evidences the significant impact of government stewardship as a form of government decision-making, support, and commitment to vaccination coverage [11,12]. The blueprint of the national-level strategy to combat the COVID-19 pandemic was shared with the states, and accordingly, the states formulated their state-specific plan to carry out vaccination coverage. Standard operating principles (SOPs) and guidelines drafted by the national and state governments such as transparent operations, real-time data collection, seamless communication with the health workers, vaccine storage, and handling helped in the optimum utilization of the resources by reducing wastage of the vaccine and, thereby, resulting in the vaccination coverage [13]. Vaccine procurement, allocation, and equitable distribution were also identified as contributing factors for vaccination coverage which involved government decision-making. Other contributing factors indicating government stewardship include collaboration with development partners, public–private health integration, financial support, and technological support.

### 1.2. Planning and Implementation

Planning and implementation have been identified as the critical contributing factor to vaccination coverage. The government in India prepared a responsive plan to ensure healthcare needs were provided by improving capacities [14]. Both the private and public healthcare sectors came together for the vaccination program to be successful. Budget planning, human resource management, training, immunization strategies, review, and monitoring were a few indicators of the process adopted by the government for planning and implementation [15]. The focus on these contributing factors by the government enabled policymakers and other private bodies to make suitable strategies revolving around the higher coverage of the population to be vaccinated [16]. Transportation, cold storage, and waste management also played a significant role in the seamless delivery of the COVID-19 vaccine [17]. Another important area in which the states worked proactively concerns the dispersal of information to create awareness around the COVID-19 vaccine [18]. At the state and district administrative level, communication strategies have played a pivotal role in the vaccination program, as it has led to phenomenal coverage and helped contain the pandemic, especially in hard-to-reach rural areas [19].

### 1.3. Community Participation

Community participation has been regarded as a prominent factor influencing vaccination coverage in past studies. Strategies based on community participation have used a well-coordinated approach in tandem with the community mobilizers [16]. As the success of the vaccination program depends upon the number of vaccinated beneficiaries, a lot of attention is given to mobilization by ensuring clear communication about the aims and objectives of the vaccination program [20]. Community participation has also been studied in the context of community engagement, especially in tribal areas where a message from the community leader or an indigenous person creates more awareness among the people [21]. To improve community participation at the primary healthcare (PHC) level, accredited social health activist (ASHA) workers contributed tremendously to educating the population and resolving their doubts related to vaccine hesitancy [22]. Additionally, the nationwide network of health centers at the national, district, and local levels, along with institutional stakeholders, aided in the dispersion of strategies about vaccine administration, transportation, distribution, dispelling of misinformation, reaching marginalized populations— transgender, street vendors, elderlies, etc. The role of community mobilizers has also been identified as significant in improving community participation, thereby, resulting in enhanced vaccination coverage [23].

### 1.4. Theoretical Framework and Hypotheses Development

**Figure 1:**
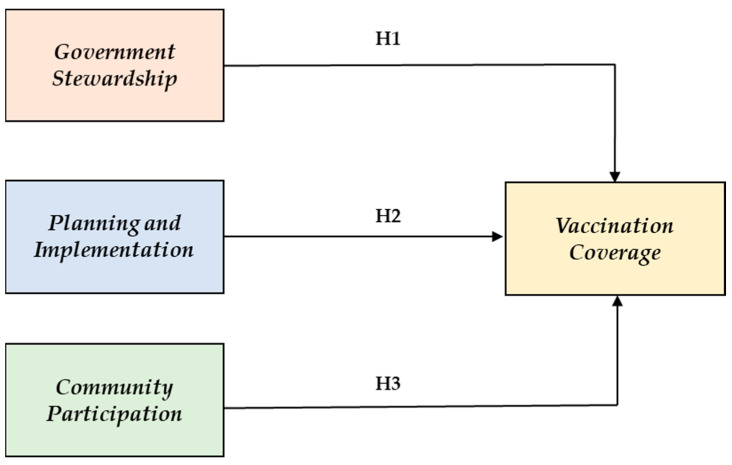
Model for empirical testing.

To develop a theoretical foundation for this study, the following hypotheses were developed based on the theoretical background described in the aforementioned subsections. This research hypotheses’ development demonstrates the relationships between the various constructs used in this study. Figure 1 illustrates the structural model designed to validate three research hypotheses evaluating the direct relationship between government stewardship, planning and implementation, and community participation with vaccination coverage.

**H1.**  *Government stewardship positively influences vaccination coverage.*

**H2.**  *Planning and implementation positively influence vaccination coverage.*

**H3.**  *Community participation directly influences vaccination coverage.*

Through this research, the intent is to highlight the learnings concerning the effectiveness of the vaccination program that was undertaken by the selected four states of the country. The relay of work can be benchmarked and customized by the practitioners and policymakers to formulate strategies and learned lessons can be tailored to other geographies in designing upcoming vaccination campaigns [24].

Therefore, the objectives of this study were two-fold as it aimed to (i) empirically examine the potential impact of government stewardship, planning and implementation, and community participation on vaccination coverage and (ii) to delineate strategic recommendations to be utilized by the vaccination program managers in the future in emerging and developing countries.

The following subsections present the theoretical background of this study and the hypotheses’ development for empirical validation.

## 2. Materials and Methods

### 2.1. Development of Research Instrument

The items in the questionnaire indicate four broad constructs described in the literature: government stewardship, planning and implementation, community participation, and vaccination coverage. Indicators of these parameters are displayed in Table 1. In the context of COVID-19 vaccination, the questionnaire items were developed and further simplified.

The survey questionnaire included twelve, twenty, seven, and three questionnaire items about government stewardship, planning and implementation, community participation, and vaccination coverage, respectively. An instance of a statement from the questionnaire characterizing administration under government stewardship is “State/National administration encouraged and facilitated streamlined work processes (such as SOPs or guidelines or strategy decisions) to leverage resource”. The questionnaire items are based on the dimensions given in Table 1.

Respondents made acceptable selections from 1 (strongly disagree) to 5 (strongly agree) for each item after reviewing the local procedures implemented during the COVID-19 vaccination program. The Likert scale, which ranges from strongly disagree (1) to strongly agree (5), was used to provide a simplified response since there are forty-two questionnaire items. To evaluate the questionnaire’s face validity, a pretest was also conducted on it. Clarity, readability, understandability, and response format accuracy were all evaluated in the pretest. Data collection was performed online.

### 2.2. Statistical Analysis

Many studies employ empirical methodology in health and policy-related research [25,26]. PLS-SEM has gained prominence in various fields from contributing in its capacity to estimate route coefficients, model latent variables under non-normality conditions, and analyze data with small to medium sample sizes [27,28]. The partial least square structured equation modeling (PLS-SEM) approach was used to analyze the proposed research model. Smart PLS 4.0, a well-known tool for PLS-SEM analysis, was utilized in this study. Alternatives to SEM include Partial Least Squares Structural Equation Modeling and Covariance-based SEM (CB-SEM). The objectives and utilization purposes of each technique are diverse; however, the two procedures are complementary [29,30].

In a public health context, the PLS-SEM technique is more suitable than the CB-SEM technique for determining correlations between important driving factors. The PLS technique investigated the causal links between constructs using the software package Smart-PLS 4.0. Attributing to the exploratory nature of the investigation, the PLS technique was employed [31]. As validated and suggested by Henseler et al. (2009), a two-step technique for data analysis was employed [32]. First, the measurement model was analyzed, and then the structural relationships between latent constructs were investigated. Before assessing the model’s structural relationship, the two-step procedure is designed to verify the measurements’ reliability and validity.

The data collected was analyzed using exploratory factor analysis (EFA) to find significant items related to the respective constructs (government stewardship, planning and implementation, and community participation), followed by confirmatory factor analysis (CFA) and structural model validation [33]. The EFA procedure was executed using the IBM SPSS 26 software package. The varimax rotation was used to optimize factor loading to improve factorability [34]. As a first step toward the factorization process, EFA, also known as the factor reduction technique [35] was carried out to extract a factor structure that conveys conceptual meaning to the overall concept of the study. A smaller subset of the overall sample was considered. With the guiding principle, the initial sample of 110 responses was considered for conducting EFA [36]. The process of reducing dimensions in EFA is iterative.

CFA was performed to validate the observed factors during the EFA quantitatively. The conceptualization of the CFA measuring model is based on EFA output. In CFA, the same factor structure was employed, but a broader sample size of 187 responses was used to validate the factors. The CFA procedure was conducted using version 26 of the SPSS-AMOS program. In addition to factorization, this study also included model testing that demonstrated the impact of government stewardship, planning and implementation, and community participation on vaccination coverage. To test the hypothesized relationships incorporated in the proposed model, Smart PLS 4.0 software was used.

### 2.3. Sampling Technique

According to standard approach for calculating sample size for studies based on the PLS-SEM technique, the size of a particular structure in the model must have a minimum of 10 times the number of structural routes [37]. Furthermore, a strong association between sample size and statistical power was documented [38]. The study suggested that 169 respondents be the minimal number needed to analyze a model made up of five exogenous variables with 80% statistical power and a 5% level of significance [37]. In the current investigation, it we made sure that these requirements were met.

### 2.4. State Selection

The states were selected based on quantitative and qualitative aspects in order to obtain a mixed representation across different geographic regions of India. The states in India have been grouped under four regions: northern, southern, western, and eastern. The quantitative parameters considered for selecting states were the percentage of partially vaccinated or fully vaccinated population and the ratio of fully vaccinated to partially vaccinated population. The analysis of vaccination status was carried out with the secondary data retrieved from the CoWIN portal (an Indian government web portal for COVID-19 vaccination registration, owned and operated by India’s Ministry of Health and Family Welfare). A few of the qualitative aspects, for instance, how the state has implemented changes in its policies to improve the uptake of the vaccines or the administrative support provided to resolve the challenges of vaccination campaigns or the variation in the speed of vaccinations were also included for the analysis.

The longitudinal vaccination coverage data were obtained from secondary sources including government records since the campaign’s inception, thus, facilitating the finalization of four states. The selected states were included in the study for the following reasons: Andhra Pradesh (southern region) for its highest immunization percentages with 61.9% for the fully vaccinated population, Himachal Pradesh (northern region) for the highest ratio of full and partial immunization (0.93) while ranking second-highest for fully vaccinated with 85% and 91% for the partially vaccinated population, Maharashtra (western region) for its vaccination reach that covered approximately 77% of its population as partially vaccinated and 57% as fully vaccinated, with a ratio of 0.74, and Odisha (eastern region) for its vaccination reach that covered approximately 77% of its population as partially vaccinated and 62% as fully vaccinated, with a ratio of 0.81.

### 2.5. Data Collection

The data were collected from 187 respondents from four states of India, namely, Andhra Pradesh, Himachal Pradesh, Maharashtra, and Odisha, directly involved in the COVID-19 vaccination campaign holding different levels of positions within the healthcare system. To collect data from respondents, a survey was conducted which lasted for four months (May 2022 to August 2022). Other qualitative characteristics, such as how the state amended the policies to improve the uptake of vaccinations, the administrative support offered to address the obstacles of the immunization campaign, and the variation in the immunization rate, are also included in the analysis.

## 3. Results

The respondents for the study were from different levels of human resources for inclusivity: namely, healthcare workers, support staff, and consulting partners. Table 2 shows the demographic profile of the respondents. The data was collected from four states of India, in which the responses from Andhra Pradesh account for 16.57%, Himachal Pradesh—33.15%, Maharashtra—23.52%, and Odisha—26.73%. Multiple designations that were similar in terms of roles and responsibilities were grouped under a broader designation term. The broad designation categories used for classifying the respondent’s designation are mentioned in Table 2. For example, the designation ‘immunization officer’ included both state and district immunization officers. Similarly, the designations ‘medical officer’, ‘chief health officer’, ‘community health officer’, ‘district health officer’, ‘block medical officer’, and ‘state medical officers’ were grouped under the umbrella term of health officers. From the total set of valid responses received, the most responses were from health officers, which accounts for 37.96%. The second largest number of responses were collected from frontline workers, which was 19.78%.

With sufficient data available for factorization, a smaller set of data was considered for EFA. Following the sequential and stepwise approach for empirical study, the factors extracted were validated through CFA. Extending the study from factorization, the proposed model was empirically validated to test the hypothesized relationships.

### 3.1. Results of Factor Analysis

In factor extraction, the factor accounting for maximum common variance is eliminated. To ensure the data sufficiency for the EFA, Kaiser-Meyer-Olkin (KMO) was observed. It was noted that the KMO reported in the analysis was 0.837, which is regarded as meritorious [39]. The process of EFA was conducted with principal component analysis as the extraction method and varimax rotation as the rotation method. To analyze convergent validity, two criteria were followed to retain the items based upon (1) no cross-loading of items and (2) factor loading to be greater than 0.5. In other words, items were deleted if the result of loadings was less than 0.5 on two or more [37]. In this study, a cut-off point of 0.5 or above was used. This value was critical in ensuring practical significance for sample sizes of 150 and above before proceeding to confirmatory factor analysis [31]. The cross-loading items were removed iteratively to improve the reliability parameters to obtain a perfect group of factors. In this iterative process, nine items were removed, which resulted in producing four factors that had an eigenvalue of more than one (refer to Table 3).

This study was executed in the following steps as proposed by Hair et al. (2021) to evaluate the obtained measurement model through IBM SPSS AMOS 26. First and foremost, Cronbach’s alpha (α) and composite reliability (CR) were utilized to assess internal consistency reliability [37]. The values of α and CR were found between 0.792 and 0.918, which is higher than the permissible threshold of 0.7 for all the factors. This indicates a satisfactory level of internal consistency reliability. Secondly, outer loadings and average variance extracted (AVE) were analyzed to gauge the convergent validity [40]. All the outer loading values were found to be equal to or greater than 0.7, whereas the AVEs values were higher than 0.5. Corresponding to these results, the convergent validity of the factors was ensured. All three values of outer loadings, α, CR, and AVE are presented in Table 4.

It was observed that the measurement model used for CFA was fitting well with the data. The analysis reported the Comparative Fit Index (CFI), Standardized Root Mean Squared Residual (SRMR), and Root Mean Square Error of Approximation (RMSEA) as 0.962, 0.064, and 0.037, respectively. The details are shared in Table 5.

Lastly, discriminant validity was also assessed through the heterotrait–monotrait ratio (HTMT), as given in Table 6. It is evident from the given Table 6 that the requirements as per HTMT were met [41]. Therefore, the results were concluded, and correspondingly, the discriminant validity of the factors was strongly supported by HTMT in the proposed model.

### 3.2. Results of PLS-SEM

**Figure 2:**
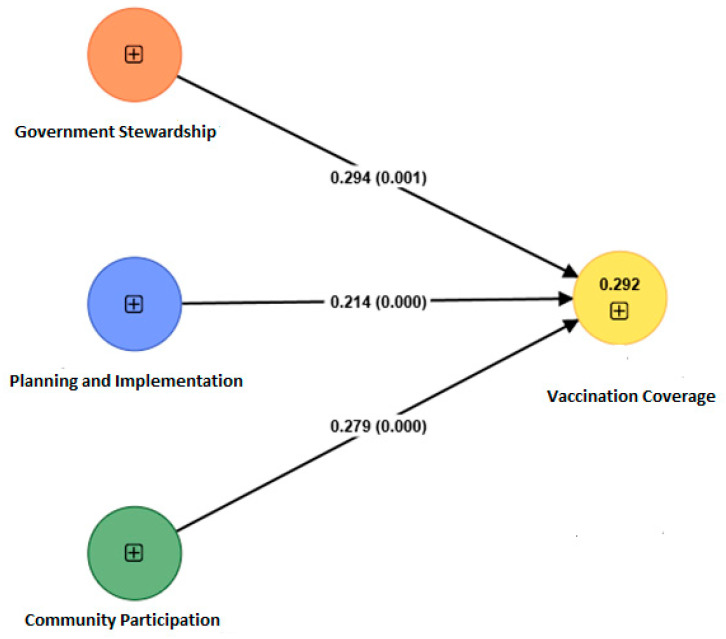
Empirical validation of the model.

The model proposed in the study exhibits three major hypotheses illustrating the impact of government stewardship, planning and implementation, and community participation on vaccination coverage. For empirical validation of the model, the data collected was used to test the hypotheses statements. The model was empirically validated with the help of SmartPLS 4.0 software. The model mapped and validated in the SmartPLS 4.0 is shown in Figure 2. The results obtained by analyzing the structural model are presented in Table 7. It was observed that all the factors were significantly influencing COVID-19 vaccination coverage. From Table 7, it is evident that the model is validated, and the proposed hypotheses are fully supported.

The model also reported 0.064 as the SRMR value, indicating a good model fit. The R-squared value of vaccination coverage obtained from the analysis is 0.294. By and large, all three relationships have β values between 0.2 and 0.3. However, the factors ‘government stewardship’ and ‘community participation’ have a greater impact on vaccination coverage in comparison with ‘planning and implementation’. The reported β values H1 and H3 are 0.294 and 0.279, respectively. In the comparison of both relationships, it is observed that the impact of government stewardship is highest, followed by community participation and planning and implementation. The reported β value for the relationship between planning and implementation and vaccination coverage is 0.214, which is the least among the three proposed hypotheses.

## 4. Discussion

The success of mass vaccination programs has been among the major concerns of healthcare practitioners and researchers. For the last three years, with the emergence of the COVID-19 pandemic, healthcare researchers, practitioners, and governments have shown their greater concerns regarding the effective allocation, utilization, and equitable distribution of vaccines. India’s COVID-19 vaccination drive claimed the position of being the fastest drive in the world. However, the major efforts to ensure that the COVID-19 vaccination drive was successful were determined by the intensity of planning and implementation of a series of events which included vaccine allocation, distribution, financial support, technological advancements, collaborations with private players, budget planning, strategizing the role of frontline workers, mobilizing the community, and removing vaccine hesitancy at the national level. In this regard, the current study examined the factors and underlying indicators that influenced vaccination coverage in India. Government stewardship, planning and implementation, and community participation were identified as the three prominent contributing factors to vaccination coverage. Previous studies have identified the practices of planning and implementing the decisions of the government in mobilizing the community to accept vaccines. This study is incremental in identifying the indicators of vaccination coverage with a micro-lens view and examining their impact on vaccination coverage by formulating a structural framework that can be used in future vaccination programs.

This study identified the customized vaccination campaign strategies, streamlining the strategic decisions in the form of SOPs and guidelines, and ensuring availability of trained human resources before vaccination programs started as the prominent indicators of government stewardship. The government encouraged and facilitated streamlined work processes to leverage resource opportunities. Additionally, each state was provided with the flexibility to customize the SOPs and guidelines for vaccination in terms of their regions and population segments. The government also showed their due concern and diligence in bringing in the technological advancements and acquiring the technical staff to implement hassle-free technical operations for vaccination programs. The results identified the integration of the public–private health infrastructure, capacity building of healthcare human resources, proactive budget planning, adequate training to the frontline healthcare workers, and conducting the capacity building sessions before the vaccination program started as the significant contributing factors of planning and implementation. The entire public and private health infrastructure was integrated for testing, tracing, and treatment of COVID-19 patients. Proactive budget planning and funds allocation was performed for different case scenarios even before the launch of the vaccination program. Furthermore, the cold chain storage capacity, to maintain COVID-19 vaccine effectiveness, was planned according to the geographical/topographic/security aspects. The opportunities were planned for people to participate in small group meetings, to be conducted in a participatory way, and focused on the topic of vaccines (vaccination schedule, benefits, and risks) before the vaccination program started in India.

This study revealed the significant indicators of community participation. The study identified that the willingness of the community to get vaccinated improved when proper information was provided to them regarding the free distribution of vaccines in their nearby centers. Active involvement of ANM, AWW, ASHA workers, vaccination team members, cold chain handlers, local influencers, prominent religious leaders, village panchayats, forest department, education department, institutions, certain people from NGOs, etc. was noted for community mobilization. The required information was made available and accessible in the public domain from multiple communication channels to dispel misinformation and improve vaccine trust and acceptance, thereby improving vaccination coverage.

To curtail the infection rate and transmission of COVID-19, government agencies responded through vaccination programs [42]. Unlike other routine immunization programs, COVID-19 immunization requires swift and immediate actions [43]. The past experiences of India in conducting mass vaccination campaigns provided a sufficient foundation, and therefore, the health workers were able to contribute in a notable way that led to exemplary planning and implementation of the COVID-19 vaccination program [44]. These findings align with the studied observations.

The study identified the pivotal role of government stewardship on immunization coverage as exhibited in Figure 2, representing the empirical relationships. The prominent indicators under government stewardship that lead to the direct impact on vaccination coverage were the integration of the public–private health sector infrastructure, frequent interaction (dialogue, meetings, presence) between the public and private healthcare sectors, handling logistics and transportation, addressing workforce shortage in many regions with challenging terrains as well as to build trust among the general population regarding the vaccine’s efficacy, safety, and affordability. This aspect of social sustainability can be achieved through tailor-made strategies for the local population that provides reassurance to them regarding vaccine safety [45]. Future vaccination initiatives may also consider accounting for using private business partners for logistics. The public–private collaboration is specifically suggested for the difficult-to-reach areas. In the event of an emergency, such as COVID-19, in recent years, the public–private partnership model can also assist in resolving workforce difficulties in other geographical regions. Creating new funding sources and studying private-sector financial methods, such as blended finance and impact investments, are equally crucial. Public funds and corporate social responsibility (CSR) can also be used in rural areas to construct vaccination programs and other primary medical care facilities. Therefore, the need to strengthen public–private partnerships has been identified to improve public healthcare systems and attract private investment in the sector operating in geographically challenging areas.

This study revealed the significant impact of community participation on vaccination coverage. The awareness among the people and mobilization initiatives were found to have a significant impact on vaccination coverage. While exploring the events for the impact of vaccine education on its coverage, as proclaimed by experts, the urban and semi-urban areas were found to have less vaccine hesitancy after communicating the information through various social media channels. These informative messages and videos dispelling vaccine hesitancy and creating confidence in populations were possible due to the digital infrastructure in urban areas. However, several rural areas in India, due to their difficult geographic terrains, remain digitally divided; hence, it became very challenging for the vaccination awareness team to improve community participation in rural and difficult-to-reach areas. Therefore, the expert opinions suggested creating and verifying community awareness through digital interventions. For this purpose, a dedicated digital infrastructure must be planned and implemented. A hybrid technical system shall be implemented which can provide the facilities of online and offline portals to address the digital divide. Due consideration has to be provided by framing national policies to improve the digital infrastructure in digitally divvied areas. The local, state, and national governments will be required to work closely for removing the digital divide and addressing inequities in future healthcare programs.

Based upon the empirical findings of this study and SWOT analysis, this study has used the recommendation matrix (Table 8) to help readers to summarize how countries or policy makers can improve performance if similar challenges for vaccination coverage might be faced in the future.

## 5. Strengths, Limitations, and Future Research Agenda

The merit of the online survey approach was that it allowed to quickly acquire information regarding diverse stakeholders’ perceptions of government stewardship, planning and implementation, and community participation from four states in India representing India comprehensively across the distinct geographies with common quantitative and qualitative characteristics related to the partially or fully vaccinated population. This study provided vaccination program managers and policymakers with the tools to examine the key variables affecting vaccination coverage. The introduction of the PLS-SEM methodology in the survey makes the findings robust, relaxes assumptions on normal distribution, and provides the ability to estimate more complex models using smaller studies, while also recommending various avenues for future research. This study provides fundamental information regarding government stewardship, planning and implementation, and community participation toward key outcomes and stakeholders involved in the COVID-19 vaccination process. Other aspects, such as innovations and flexibility of the procurement and delivery system of vaccinations must be further explored in future qualitative studies with the help of grounded theory. Future studies can also identify other indicators of public health outcomes and measure the impact of government stewardship, planning and implementation, and community participation on public health outcomes at large and extending to a wider range of geographies in India.

An unequal distribution of participants from various states was limited due to the online survey form and participant self-selection. To comprehend the perspectives, thoughts, and opinions of a diverse set of respondents, online surveys should be followed by or supplemented with other research designs, such as in-depth qualitative investigations. Future research should employ more stringent sampling techniques to ensure an equal representation across various respondent profiles as represented in a nationally conducted household survey study [46].

## 6. Conclusions

The present study’s premise was based upon the backdrop of two research objectives. Firstly, this study aimed to empirically examine the potential impact of government stewardship, planning and implementation, and community participation on vaccination coverage. For this purpose, the data were collected from four different states including the northern, eastern, western, and southern regions of India. The study employed structural equation modeling to analyze the hypothesized relationships. The results confirmed the significant impact of government stewardship on vaccination coverage, followed by community participation and planning and implementation.

Secondly, the study aimed to delineate strategic recommendations to be utilized by vaccination program managers in the future in emerging and developing countries. Various strategic recommendations have been provided based on the findings of the study that shall be useful for program managers, policymakers, and public health officials in future vaccination programs. The identified factors and their relevant relationship with vaccination coverage will not only help the program managers of India but also serve as a successful operational model for vaccination programs in other parts of the world.

## Figures and Tables

**Table 1:** Dimensions of the framework for immunization coverage.

S. No.	Dimensions	Subfactors
1	Government Stewardship	Administrative decision-making
2		SOPs and guidelines
3		Development partners’ support
4		Financial support
5		Vaccines procurement and allocation management
6		Technology support
7		Public–private health sector integration
8	Planning and Implementation	Health infrastructure
9		Budget planning
10		Human resource management
11		Vaccine and ancillary supplies demand forecasting
12		Vaccine supply chain, logistics, and storage
13		Social and Behavior Change Communication (SBCC) strategies
14		Immunization campaign strategies
15		Review and monitoring
16		Interdepartment collaboration
17	Community Participation	Mobilization initiatives
18		Awareness generation—demand generation and vaccine hesitancy
19		Community mobilizers

**Table 2:** Respondents’ profile.

States	N = 187	Percentage
Andhra Pradesh	31	16.57
Himachal Pradesh	62	33.15
Maharashtra	44	23.52
Odisha	50	26.73
Designation		
Immunization officer	27	14.43
Health officer	71	37.96
Frontline health workers	37	19.78
IEC officer	2	1.06
Development partner officer	3	1.60
Cold chain manager	17	9.09
Vaccination logistics manager	14	7.48
Program Manager	4	2.13
Technical support staff	4	2.13
Others	8	4.27

**Table 3:** Results of EFA.

Items	Factors			
	Government Stewardship	Planning and Implementation	Community Participation	Vaccination Coverage
State administration encouraged and facilitated streamlined work processes (such as SOPs or guidelines or strategies decisions) to leverage resource opportunities.	0.898	-	-	-
Region-specific and population group-specific immunization campaign strategies were implemented by the state administration.	0.746	-	-	-
State administration was able to attract and retain its qualified scientific-technical staff and absorb key technologies.	0.714	-	-	-
COVID-19 vaccine allotment and distribution were adequately carried out with respect to all the districts.	0.699	-	-	-
Development partners provided proprietary information or suggestions to the state administration.	0.691	-	-	-
COVID-19 vaccine procurement was conducted with sufficient frequency with tolerable time duration.	0.667	-	-	-
Customized immunization campaign strategies helped to reduce COVID-19 vaccine hesitancy.	0.641	-	-	-
The standard operating procedure (SOPs) and guidelines related to COVID-19 were adequately available (such as administration, health professionals, and beneficiaries).	0.639	-	-	-
State administration encouraged technological initiatives.	0.639	-	-	-
There was frequent interaction (dialogue, meetings, presence) between the public and private healthcare sectors.	0.634	-	-	-
State administration was able to generate innovative and advanced technological processes (such as online portals or mobile applications or social media) to support COVID-19 immunization coverage for various stakeholders.	0.606	-	-	-
The entire public and private health infrastructure was integrated for testing, tracing, and treatment of COVID-19 patients.	-	0.917	-	-
The cold chain storage capacity, to maintain COVID-19 vaccine effectiveness, was planned according to the geographical/topographic/security aspects.	-	0.781	-	-
Capacity augmentation of healthcare sector human resources was achieved.	-	0.734	-	-
Adequate training and technology update sessions were conducted.	-	0.729	-	-
Sufficient information on vaccines was available (consider both health and non-health sources).	-	0.726	-	-
Proactive budget planning and funds allocation were conducted for different scenarios before the launch of immunization.	-	0.713	-	-
There were opportunities for people to participate in small group meetings, conducted in a participatory way, and focused on the topic of vaccines (vaccination schedule, benefits, and risks).	-	0.679	-	-
The cost of the COVID-19 vaccine through the supply chain network to service delivery points was reviewed frequently.	-	0.657	-	-
Vaccines and ancillary supplies were adequately and regularly forecasted based on the past days’ immunization data.	-	0.643	-	-
Adequate healthcare human resources were deployed.	-	0.631	-	-
People were willing to immunize themselves when the free vaccine was made available nearest to their locality.	-	-	0.929	-
Community mobilization through Auxiliary nurse midwife (ANM), Anganwadi Helpers (AWW), ASHA workers, vaccination team members, cold chain handlers, local influencers, prominent religious leaders, village panchayats, forest department, education department, institutions, people from NGOs, etc.	-	-	0.821	-
Interdepartment collaboration was involved so that people helped each other in planning and provided suggestions.	-	-	0.808	-
There was availability and accessibility of information in the public domain from multiple communication channels to dispel misinformation.	-	-	0.801	-
Interdepartment collaboration was involved to help the state administration/private organizations/institutions.	-	-	0.797	-
People were willing to immunize since the government, doctors, public figures, family, and friends encouraged them to take COVID-19 vaccinations.	-	-	0.752	-
People were well-informed about COVID-19 immunization and the benefits of vaccination.	-	-	0.738	-
People were willing to immunize if they have young children or elderly parents at home.	-	-	0.711	-
People were aware of the cause, symptoms, and treatment protocols related to COVID-19 disease.	-	-	0.699	-
There was a high impact on people’s participation in COVID-19 immunization coverage performance in the state.	-	-	-	0.765
There was a high impact of state administration on COVID-19 immunization coverage performance in the state.	-	-	-	0.657
There was a high impact of the planning aspect on COVID-19 immunization coverage performance in the state.	-	-	-	0.599

**Table 4:** Outer loadings, Cronbach’s alpha, CR, and AVE.

Factors	Items	Outer Loadings	Cronbach’s Alpha (α)	Composite Reliability (CR)	Average Variance Extracted (AVE)
	GS1_1	0.996	0.918	0.92	0.516
	PI14_1	0.718			
	GS6_2	0.702			
	GS5_2	0.675			
	GS3_2	0.682			
Government stewardship	GS5_1	0.674			
	PI14_2	0.659			
	GS2_1	0.705			
	GS1_2	0.685			
	GS7_2	0.659			
	GS6_1	0.680			
	PI8_1	0.989	0.903	0.901	0.508
	PI12_3	0.705			
	PI8_3	0.707			
	PI10_3	0.652			
Planning and implementation	PI13_1	0.641			
	PI9_1	0.706			
	PI13_2	0.692			
	PI12_2	0.643			
	PI11_2	0.607			
	PI10_1	0.986			
	CP17_1	0.695	0.897	0.906	0.495
	CP19_1	0.653			
	PI16_2	0.656			
	CP18_3	0.672			
Community participation	PI16_1	0.644			
	CP17_2	0.634			
	CP18_2	0.654			
	CP17_3	0.691			
	CP18_1	0.683			
	VC20_3	0.661	0.792	0.812	0.598
Vaccination coverage	VC20_1	0.950			
	VC20_2	0.673			

**Table 5:** Model fit indices.

Measure	Estimate	Threshold	Interpretation
CMIN/DF	1.258	Between 1 and 3	Excellent
CFI	0.962	>0.95	Excellent
SRMR	0.064	<0.08	Excellent
RMSEA	0.037	<0.06	Excellent
PClose	0.992	>0.05	Excellent

**Table 6:** Discriminant validity assessment using the HTMT test.

	Government Stewardship	Planning and Implementation	Community Participation	Vaccination Coverage
Government Stewardship				
Planning and implementation	0.235			
Community participation	0.295	0.009		
Vaccination coverage	0.47	0.351	0.311	

**Table 7:** Hypothesis testing.

Hypothesis	Path	β	T Statistics	*p*-Values
H1	Government stewardship → Vaccination coverage	0.294	3.463	0.001
H2	Planning and implementation → Vaccination coverage	0.214	3.652	0.000
H3	Community participation → Vaccination coverage	0.279	3.880	0.000

**Table 8:** SWOT analysis-based recommendation matrix.

Recommendation Matrix for Improving Vaccination Coverage	Opportunities	Threats
**Strengths**	Using Strengths to Maximize Opportunities Customized vaccination campaign strategies Streamlining the strategic decisions in the form of SOPs and guidelines Acquiring the scientific-technical staff before vaccination programs started Encouraging streamlined work processes to leverage resource opportunities Using multiple communication channels to improve vaccine acceptance among mass population	Using Strengths to Minimize Threats Integration of public–private health infrastructure Capacity building of healthcare human resources Adequate training to the frontline healthcare workers Conducting the technology update sessions before the vaccination program Integration of public and private health infrastructure for testing, tracing, and treatment of COVID-19 patients.
**Weaknesses**	Using Opportunities to Minimize Weaknesses Creating and verifying community awareness through digital interventions Using hybrid technical system Achieving social sustainability through tailor-made strategies for the local population Creating new and sustainable funding sources through public sector A 360-degree active involvement of multi-level stakeholders for community mobilization	Prevent Weaknesses turning to Threats Encouraging multi-stakeholder partnerships Transforming vaccine hesitancy into vaccine confidence Improving the inclusiveness of vulnerable and marginalized populations Ensuring equitable access via introducing innovative interventions Adopting innovative approaches in public health system Framing national policies to improve the digital infrastructure in digitally divvied areas

## Data Availability

Not applicable.
